# Serious Adverse Drug Reactions to COVID-19 Vaccines in the Pediatric Population: A Retrospective, Cross-Sectional Study Utilizing the Eudravigilance Database for the European Economic Area

**DOI:** 10.3390/jcm14186542

**Published:** 2025-09-17

**Authors:** Grzegorz Nazar, Julia Olszlegier, Aleksandra Kamińska, Katarzyna Plata-Nazar, Wojciech Nazar

**Affiliations:** 1 Faculty of Medicine, Medical University of Gdańsk, Marii Skłodowskiej-Curie 3a, 80-210 Gdańsk, Poland; grzegorz.nazar@gumed.edu.pl (G.N.); olszlegierj@gmail.com (J.O.); aleksandra.kaminska@gumed.edu.pl (A.K.); 2 Department of Pediatrics, Gastroenterology, Allergology and Nutrition, Medical University of Gdańsk, Nowe Ogrody 1-6, 80-803 Gdańsk, Poland; knazar@gumed.edu.pl; 3 Laboratory of Experimental and Translational Allergology, Department of Allergology, Medical University of Gdańsk, Mariana Smoluchowskiego 17, 80-214 Gdańsk, Poland

**Keywords:** COVID-19, Comirnaty, Spikevax, vaccination, adverse drug reactions, real-world, EudraVigilance

## Abstract

**Background:** During the global fight against the COVID-19 pandemic, vaccinations have been widely recognized as the most effective and generally safe method for preventing the spread of COVID-19. However, it has been reported that children may experience post-vaccination serious adverse drug reactions (SADRs). Thus, we aimed to analyze the risk of SADRs to COVID-19 vaccines in the pediatric population. **Methods:** In this retrospective, cross-sectional study, 5422 cases of SADRs (n = 5018 for Pfizer BioNTech, Comirnaty and n = 494 for Moderna, Spikevax) were analyzed after 37,344,343 doses of COVID-19 vaccines were administered. This study covered the European Economic Area. The analysis period for both vaccinations and SADRs spanned from 7 December 2020 to 5 October 2023. The analysis encompassed 207 types of SADRs grouped into 12 categories. All estimated real-world reporting rates were reported as normalized per million ADR reports and adjusted using real-world trial-based scaling (APMR). **Results:** The total estimated real-world reporting rates of SADRs were 5792 APMR for Comirnaty and 5671 for Spikevax. The most commonly reported clinical categories of suspected SADRs for both vaccines were neuropsychiatric, cardiovascular and gastroenterological disorders. The most often reported SADRs encompassed headaches, myocarditis, episodes of syncope, dizziness and dyspnea. **Conclusions:** According to the data from this study, several SADRs were reported in children following COVID-19 vaccination. The estimated real-world reporting rates of SADRs related to COVID-19 vaccines seem to be rare among children. Additionally, the data suggest that Comirnaty (Pfizer-BioNTech) may have a similar risk profile compared to Spikevax (Moderna).

## 1. Introduction

COVID-19, caused by the SARS-CoV-2 virus, presents a significant public health challenge due to its complex pathophysiology and varied clinical manifestations [1,2]. The virus primarily enters human cells via the ACE2 receptor, initiating a cascade of immune responses that can lead to systemic inflammation and multi-organ dysfunction [1,2,3,4]. Notably, severe cases may progress to acute respiratory distress syndrome (ARDS), characterized by widespread alveolar damage and hypoxemia, which is a leading cause of mortality in COVID-19 patients [1,2].

Beyond respiratory complications, COVID-19 has been associated with an increased risk of cardiovascular events, including heart attacks and strokes [5,6,7]. Additionally, the emergence of post-acute sequelae, commonly referred to as “Long COVID,” has underscored the prolonged impact of the disease, with patients experiencing persistent symptoms affecting various organ systems [3,4].

Treatment strategies for COVID-19 have evolved to include antiviral agents like remdesivir and nirmatrelvir-ritonavir (Paxlovid), which have demonstrated efficacy in reducing disease severity and hospitalization rates when administered early in the course of infection [8,9,10,11]. Immunomodulatory therapies, such as dexamethasone and baricitinib, are employed to mitigate the inflammatory response in severe cases [8,9,10,11].

Despite these advancements, the most effective measure remains prevention through vaccination, which has been shown to significantly reduce the incidence of severe disease, hospitalization, and death associated with COVID-19 [12,13,14,15]. Vaccinations have a profound impact on individuals, society, and the economy by promoting herd immunity and significantly reducing hospitalization and mortality rates. Large clinical trials have proven that the benefit of active immunization clearly outweighs the risk of potential adverse drug reactions (ADRs) [16,17,18,19]. The collective immunity not only protects those vaccinated but also safeguards vulnerable populations, lessens healthcare burdens, and contributes to economic stability by reducing pandemic-related disruptions [20].

However, it has been reported that some individuals, including children, may experience post-vaccination ADRs [17,18,21]. Further on, vaccines are among the most commonly reported drugs causing ADRs [22]. The European Medicines Agency (EMA) collects and analyses data on suspected ADRs to medicines that have been authorized or are in clinical trials in the European Economic Area (EEA), including COVID-19 vaccines [23]. The following categories of ADR frequency are distinguished: very common (≥1/10); common (≥1/100 to <1/10); uncommon (≥1/1000 to <1/100); rare (≥1/10,000 to <1/1000); very rare (<1/10,000). The EMA defines an ADR as a noxious and unintended response to the medicine [23].

EMA also further distinguishes a serious ADR as ‘a reaction that corresponds to any untoward medical occurrence that a tiny dose results in death, is life-threatening, requires inpatient hospitalization or prolongation of existing hospitalization or results in significant disability [23].

The EMA has authorized three COVID-19 vaccine formulations for use in children. Comirnaty (Pfizer-BioNTech) and Spikevax (Moderna) are approved for children aged 6 months and older, while Nuvaxovid (Novavax) is authorized for use in children aged 12 years and above [24,25].

The Summary of Product Characteristics (SmPC) for all three of those formulations indicates the obligation of additional monitoring of the ADRs and the suspicions of ADRs [26,27,28]. Further on, children may be more susceptible to certain adverse effects due to the immaturity of absorption, metabolism, and drug elimination mechanisms [29]. This highlights the importance of tracking ADRs specific to children. Monitoring of ADRs that are prevalent in children is essential to ensure the safety of medications administered in pediatric populations. 

Studies conducted on COVID-19 disease during the early stages of the pandemic suggested that children and adolescents suffer mostly mild manifestations of the SARS-CoV-2 infection [30,31], and therefore were classified by the WHO as the group of lower priority for the COVID-19 immunizations [21,32]. As a result, children began receiving COVID-19 vaccinations relatively late. Combined with limited data on this age group during the vaccine development process, this delay has contributed to a gap in comprehensive data analyses on potential ADRs to authorized COVID-19 vaccines in children [29].

The overwhelming majority of ADRs to the COVID-19 vaccine are mild and include headache, pyrexia and vomiting [15,33]. Severe post-vaccination ADRs are rare and have been reported in children mostly through case reports and analysis of smaller groups [21,31]. For example, there have been reports of anaphylaxis secondary to the mRNA COVID-19 vaccine [31,34]. Other severe ADRs include multisystem inflammatory syndrome, respiratory failure, and cardiovascular dysfunction [21,35].

However, so far, in the pediatric population analyses of crude counts of reported ADRs have been conducted [15,21,36]. Moreover, the majority of the studies report mild to moderate ADRs and the more clinically relevant serious ADRs remain less understood. Additionally, most existing studies focus on specific groups of potential ADRs, rather than encompassing a wide range of clinical specialties.

Therefore, there is a need for large-scale studies to monitor the population and accurately estimate the real-world reporting rates of potential serious ADRs (SADRs) following COVID-19 vaccination. Such research based on real-world data will provide valuable insights into the vaccines’ safety profile and risk-benefit ratio in pediatric populations. A deeper understanding of COVID-19 vaccine pharmacovigilance in children will ultimately lead to a reduced number of hospitalizations, intensive care unit admissions and deaths.

## 2. Aim

We aimed to analyze the risk of serious adverse drug reactions to COVID-19 vaccines in the pediatric population.

## 3. Methods and Materials

This retrospective, observational, cross-sectional study was conducted in accordance with the Strengthening the Reporting of Observational Studies in Epidemiology Statement: Guidelines for Reporting Observational Studies (STROBE) [37].

### 3.1. Data Acquisition—EudraVigilance Database

**Figure 1:**
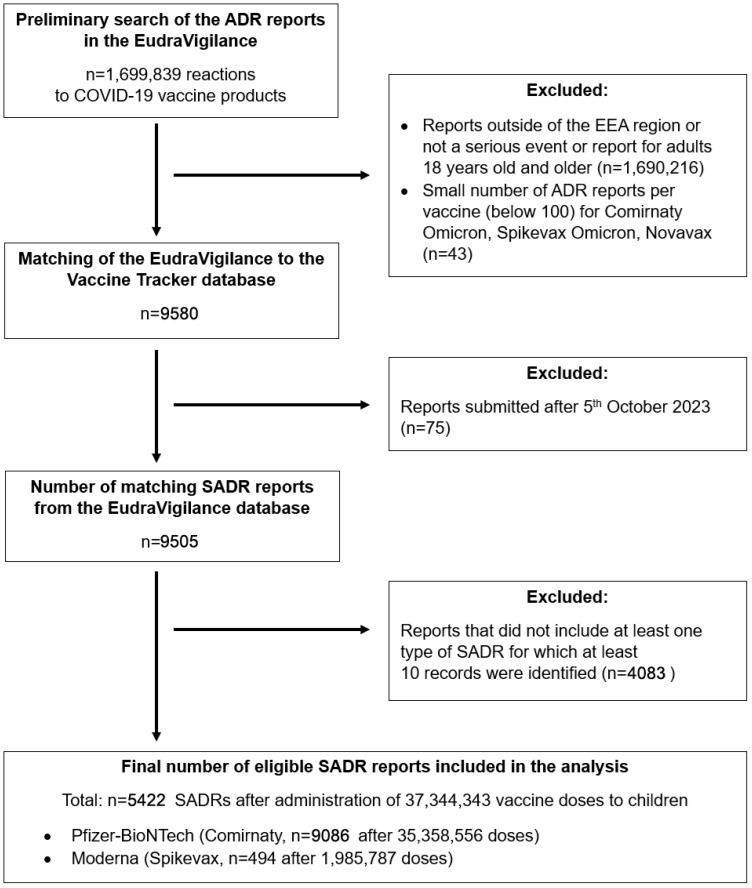
Study design. The numbers of ADRs correspond to anonymized records of patient cases reporting ADRs.

On 17 October 2024, a preliminary search of the EudraVigilance database was performed to find suspected ADRs to three COVID-19 vaccines that were administered to children in the EEA: Comirnaty, Spikevax and Novavax, including the anti-Omicron variants. No filters were used in the initial search, resulting in a total of 1,699,839 spontaneously reported, anonymized records of patient cases reporting ADRs associated with COVID-19 vaccine products (Figure 1). Access to the database was limited to open-access data and comprised descriptions of the ADRs along with restricted demographic information, including age group, sex, and primary source qualification.

EudraVigilance serves as the central system for collecting, managing, and analyzing reports of suspected ADRs for medicines authorized in the EEA [23,38]. It is the largest and most comprehensive pharmacovigilance database in Europe, containing reports submitted by healthcare professionals, patients, marketing authorization holders, as well as data from clinical studies and post-marketing surveillance. We selected this source because, to the authors’ knowledge, it represents the most credible and authoritative repository of ADR data in the EEA. Its scope, recognition, and governance by the EMA ensure high standards of data collection and reporting, which makes it widely accepted by regulators, researchers, and clinicians.

The use of EudraVigilance provides several strengths that enhance the robustness of pharmacovigilance studies. Its extensive size and geographical coverage ensure representation of a large and diverse patient population across all EEA member states. Reports are coded using the internationally standardized Medical Dictionary for Regulatory Activities (MedDRA) terminology, which guarantees consistency in classification and facilitates cross-study comparisons [39]. Importantly, the database is publicly accessible in aggregated form, allowing transparency and enabling independent scrutiny of drug safety profiles. These characteristics collectively seem to make EudraVigilance one of the most reliable systems for capturing suspected ADRs in Europe.

Nevertheless, several limitations inherent to spontaneous reporting databases must be acknowledged. The most important is underreporting, as not all adverse reactions are documented, particularly if they are mild, well known, or not easily recognized as drug-related [40]. Reporting bias may also occur, with rare or severe reactions more likely to be submitted, or in situations of heightened public or media attention. Moreover, the database records suspected associations rather than proven causality; therefore, signals generated from EudraVigilance require further epidemiological or clinical validation. Finally, despite built-in mechanisms to minimize redundancy, duplicate reporting cannot be fully excluded, which may inflate case counts for certain ADRs.

### 3.2. Data Acquisition—ECDC Vaccine Tracker Database

Moreover, to obtain data on the number of administered vaccine doses in the pediatric population in the EEA, the ECDC Vaccine Tracker database was searched to identify eligible records. The data were provided on a weekly basis, covering the period from the 7 December 2020 to the 5 October 2023. Since 5 October 2023, the ECDC Vaccine Tracker has not collected any new data. During this time, about 60 million doses of approved vaccines were administered to children across the EU/EEA.

### 3.3. Data Filtering and Processing

Any records of SADRs from the EudraVigilance that were reported outside the EEA region were excluded from the study. Thus, the region of the EudraVigilance databased matched the region of the ECDC database. Moreover, since the records of serious events are usually more consistently reported, EudraVigilance records that did not have a category of SADR were also removed from further analysis. In addition to that, records that did not describe SADRs in children (persons under 18 years old) were eliminated from the evaluation. In total, n = 1,690,216 were excluded.

Furthermore, from 9580 records that were further processed, for Comirnaty Omicron, Spikevax Omicron and Novavax only 43 reports matching the previous criteria (seriousness of the event, region and population) were identified. These records were also excluded from the analysis due to an insufficient overall sample size. Next, any reports added to the EudraVigilance database after 5 October 2023, were excluded (n = 75). As a result, the EudraVigilance database aligned with the timeframe (from 7 December 2020, to 5 October 2023) and the region (EEA) of the ECDC database.

In total, 9505 records for the two vaccines approved from the pediatric population were included in the analysis: Comirnaty (Pfizer-BioNTech, Tozinameran, n = 9086) and Spikevax (Moderna, Elastomeran, n = 494). The total number of administered vaccine doses for eligible vaccines was 37,344,343 (35,358,556 doses for Comirnaty and 1,985,787 doses for Spikevax).

Subsequently, data on all available types of serious adverse drug reactions (SADRs) and their corresponding clinical categories (system organ classes) were extracted from the EudraVigilance database (n = 13,056 types of SADRs across 27 clinical categories—Supplementary Material S1). To concentrate on the clinical manifestations of suspected SADRs, only those classified as “disorders” related to various human body systems—such as “neurological disorders” or “hematological disorders”—were included in the further analysis. Conversely, groups of SADRs that (1) were overly broad; (2) were unlikely to be complications arising from COVID-19 vaccination (indicating an improbable causal relationship); or (3) involved SADRs not directly linked to clinical post-vaccination symptoms, were eliminated from the analysis: “injury, poisoning, and procedural complications”, “investigations”, “general disorders and administration site conditions”, “infections and infestations”, “product issues”, “surgical and medical procedures” as well as “social circumstances”. The total number of excluded types of SADRs was n = 7731.

The remaining 20 categories were consolidated into 12 broader groups (Supplementary Material S1). Furthermore, only those types of SADRs with at least 10 reported instances were considered for evaluation (n = 207). Records of suspected SADRs that included at least one of these 207 types were incorporated into the final assessment, resulting in n = 5422 (Figure 1).

### 3.4. Normalization Per ADR Report

To allow comparison across vaccines and ADR types, ADR counts were normalized by the total number of ADRs reported for each vaccine. Normalized reporting rates were scaled to per million ADR reports, so that each ADR’s reporting rates reflects its relative contribution to all reported ADRs for that vaccine.

### 3.5. Trial-Adjusted Scaling

Normalized ADR reporting rates were adjusted to match clinical trial rates, using a uniform ADR rate of 1% for all ADRs for both studied vaccines (adjusted per million reports, APMR). These estimated real-world reporting rates correspond to the estimated total frequency of grade 3 and grade 4 reactions as reported in the real-world vaccine registration trials (phase 2/phase 3 trials) [19,41,42,43,44,45]. This scaling provides an approximate expected reporting rate of each ADR if clinical trial rates are applied to real-world data, accounting for asymptomatic cases.

Of note, the APMR-based values represent reporting activity within the spontaneous reporting system and do not reflect the true frequency or incidence of SADRs, as spontaneous reports are subject to under-reporting, reporting biases, and incomplete exposure data.

### 3.6. Statistical Analysis

Categorical data were described through counts and proportions. Moreover, 95% confidence intervals (95% CIs) for the estimated real-world reporting rates were calculated using a binomial model based on trial sample sizes (n = 6199 for Comirnaty; n = 10,288 for Spikevax, according to the cohorts from relevant registration trials for children) [19,41,42,43,44,45]. Lower and upper CI bounds were scaled proportionally to the estimated real-world reporting rates of ADRs.

As this study is descriptive in nature, no *p*-values were calculated, and all analyses are presented solely in a descriptive manner without statistical comparisons.

All statistical analyses and machine learning modeling were conducted in Python 3.10 (Python Software Foundation, Beaverton, OR, USA, 2021) using the Pandas library (version 2.1.3; The Pandas Development Team, NumFOCUS, Austin, TX, USA, 2023) and the Scikit-learn library (version 1.2.1; Scikit-learn Developers, Inria, Paris, France, 2023).

## 4. Results

In total, 37,344,343 administered doses of COVID-19 vaccines in the pediatric population were recorded, comprising 35,358,556 doses of Comirnaty and 1,985,787 doses of Spikevax, according to data from the EudraVigilance database. From the Vaccine Tracker database, 5422 reported cases of SADRs met the inclusion criteria for this study. Of these, 5018 cases were associated with Comirnaty and 494 with Spikevax (Figure 1 and Supplementary Material S1). Reports originating from healthcare professionals accounted for 50.4% of SADRs for Comirnaty and 57.9% for Spikevax (Table 1).

### 4.1. General Characteristics

The sex distribution of affected children was comparable between the two vaccines, with males representing 49.5% of Comirnaty cases and 50.5% of Spikevax cases. However, the sex of the patient was not reported in 2.0% of Comirnaty cases and 0.7% of Spikevax cases (Table 1).

Age stratification of the eligible SADRs demonstrated that in the 0–1 month age group, 1.1% of cases were linked to Comirnaty and 3.5% to Spikevax. In the 2 months–2 years age group, Comirnaty accounted for 1.3% of SADRs, while Spikevax accounted for 1.0%. In the 3–11 years age group, Comirnaty exhibited a notably higher proportion of SADRs (10.7%) compared to Spikevax (1.5%). The highest proportion of SADRs was observed in the 12–17 years age group, with 86.9% of cases for Comirnaty and 94.1% for Spikevax.

### 4.2. Estimated Real-World Reporting Rates of SADRs

All estimated real-world reporting rates were reported as normalized per million ADR reports and adjusted using real-world trial-based scaling (APMR). Importantly, the APMR-based values represent reporting activity within the spontaneous reporting system and do not reflect the true frequency or incidence of SADRs.

The sum of analyzed SADRs was 11,802. The total estimated reporting rate of SADRs was 5792 APMR for Comirnaty and 5671 for Spikevax (Table 2). Thus, for both vaccines, the risk of any serious ADR was about 0.57% (about 5000 per million).

For Comirnaty, the most often reported ADRs were headache with 437 APMR compared to 592 APMR for Spikevax, myocarditis with 332 APMR compared to 419 APMR for Spikevax, syncope with 265 APMR compared to 279 APMR for Spikevax, dizziness with 217 APMR compared to 206 APMR for Spikevax, and dyspnea with 199 APMR compared to 239 APMR for Spikevax.

### 4.3. Estimated Real-World Reporting Rates of SADR Categories

The reported ADRs were grouped into 12 clinical categories (Table 3). The allocation of specific ADRs to these categories is outlined in Supplementary Material S1. For Comirnaty, the most often reported categories of SADRs were neuropsychiatric disorders (1239 APMR), cardiovascular disorders (853 APMR), and gastroenterological disorders (514 APMR). A similar trend was observed for Spikevax, with estimated rates of 1263 APMR, 938 APMR, and 505 APMR, respectively.

Overall, compared to Spikevax, Comirnaty was associated with higher reporting rates of ADRs in certain categories, including hemato-oncological disorders (Comirnaty vs. Spikevax: 144 vs. 106 APMR) and obstetrical and gynecological disorders (137 vs. 86 APMR). Conversely, Comirnaty showed lower estimated reporting rates of ADRs than Spikevax in categories such as musculoskeletal and connective tissue disorders (345 vs. 419 APMR), renal and urinary disorders (32 vs. 66 APMR), and congenital, familial, and genetic disorders (5 vs. 27 APMR).

## 5. Discussion

In this retrospective, cross-sectional study of more than 5400 cases of suspected SADRs reported after administering over 37,000,000 COVID-19 vaccine doses, it seems that (1) overall, in children the estimated real-world reporting rates of SADRs to COVID-19 mRNA vaccines appears to be rare with a rate of about 0.57%; (2) compared to Spikevax (Moderna), vaccination with Comirnaty (Pfizer-BioNTech) seems to be associated with a very similar risk of SADRs; (3) the most often reported categories of SADRs were neuropsychiatric, cardiovascular and gastroenterological disorders (Table 3, Supplementary Material S2).

The estimated real-world reporting rates represent reporting activity within the spontaneous reporting system and do not reflect the true frequency or incidence of SADRs, as spontaneous reports are subject to under-reporting, reporting biases, and incomplete exposure data.

### 5.1. Comparison to EMA’s Data

It can be observed that in the EMA’s product characteristics for both vaccines, the adverse reactions classified as very common, common, and uncommon tend to represent less severe conditions compared to those categorized as rare and very rare [27,28]. When comparing our findings with EMA data, myocarditis and pericarditis—among the most severe post-vaccination events—are likewise classified as rare for both vaccines [27,28].

These life-threatening conditions can be accompanied by more usual symptoms, such as dizziness, headache, arthralgia, nausea and myalgia, which were also identified as some of the most common reactions in our study. However, based on our results and following the EMA’s classification, their estimated real-world reporting rate remains uncommon or rare [46].

On the other hand, in EMA’s reports, these accompanying reactions are considered common or even very common. The discrepancies between the EMA’s characteristics and our findings may arise from the fact that the EMA’s product characteristics encompass both children and adults and include both severe and non-severe ADRs, whereas our study specifically focuses on severe ADRs in the pediatric population. Moreover, in EMA’s characteristics, the severity grades of the reactions are not reported, just the frequencies, which may also explain the differences between our study’s estimated rates and EMA’s data.

In addition to that, we analyzed SADRs in 4 age groups: 0–1 month, 2 months–2 years, 3–11 years and 12–17 years age group. The percentage of reported SADRs was higher in the 12–17 years age group (86.9% of cases for Comirnaty and 94.1% for Spikevax) than in the remaining groups (Table 1). This may be explained by the fact that the age group 12–17 was the first pediatric group to receive vaccination recommendations following EMA’s approval for administration (consecutively Comirnaty on 28 May 2021 and Spikevax on 23 July 2021, which likely caused most reports involving this group [47,48].

EMA recommended approval of usage of Comirnaty in children from 5 to 11 on 25 November 2021 and of Spikevax on 24 February 2022. For children older than 6 months of age both vaccines were advised on 19 October 2022 [24].

### 5.2. Comparison to the VAERS Database

It must be emphasized that direct comparisons between our estimated rates of SADRs from the EU-derived EudraVigilance database and US-based VAERS data are limited, as the former are expressed as estimated real-world reporting rates per million ADR reports and were adjusted using real-world trial-based scaling, whereas VAERS reflects real-world, spontaneously reported events [49,50,51,52]. Nonetheless, VAERS provides the most appropriate reference available, as it is also a retrospective, self-reported, voluntary database of ADRs to COVID-19 vaccines in the US, while our dataset originates from the European pharmacovigilance system. According to VAERS, the risk of any SADR, defined as a “SADR requiring medical care,” was approximately 0.9–1.1% for children aged 5–11 years, 0.5–0.8% for adolescents aged 12–17 years, and 0.6–0.9% for booster doses [49,50,51,52]. Hospital admissions were reported at 0.57%, 0.2–0.3%, and 0.1–0.4%, respectively. In comparison, the estimated risk of any serious ADR in the EudraVigilance database is approximately 0.57% (about 5000 per million), falling between the VAERS estimates for ADRs that “needed medical care” and those resulting in hospitalization [49,50,51,52].

These observations suggest that, despite differences in reporting systems and methodology, the estimated rates of serious ADRs in the EU are rather consistent with US data. However, differences in healthcare access, reporting behavior, and pharmacovigilance practices should be considered when interpreting these comparisons.

### 5.3. Comparison to Vaccine Registration Trials

Furthermore, the estimated risk of serious adverse events reported in phase 2 and phase 3 registration trials ranged from approximately 0.5% to 1.5% [19,41,42,43,44,45]. In comparison, the overall risk observed in our study (0.57%) falls well within this range, suggesting a consistency between real-world pharmacovigilance data and the findings from randomized controlled trials [19,41,42,43,44,45]. This alignment provides additional reassurance regarding the potential generalizability of the data, indicating that the incidence of serious adverse reactions observed under controlled trial conditions seems to reflect patterns detected in post-marketing retrospective surveillance.

### 5.4. Estimated Real-World Reporting Rates of SADR Categories

Notably, inconsistencies in the nomenclature of ADRs were observed, such as the classification of abdominal discomfort under four distinct terms: ‘abdominal pain,’ ‘abdominal pain lower,’ ‘abdominal pain upper,’ and ‘abdominal discomfort’ (Supplementary Material S2). These discrepancies may lead to inaccurate reporting and data bias, thereby underestimating the reporting rates of certain symptoms. To address this issue, we introduced clinical categories of ADRs, consolidating similar symptoms into unified groups. This approach enhances the accuracy and consistency of reporting while mitigating data fragmentation.

Alessia Zinzi et al. described ADRs following immunization with Comirnaty and Spikevax in children aged 5–11, using data from the EudraVigilance database [15]. In this study, the authors did not differentiate between serious and non-serious ADRs, nor did they specify which vaccine was administered to each individual. The categories of adverse reactions were ranked in the following order, from most to least common: general disorders and administration site conditions (28.9% of all ADRs), nervous system disorders (16.4%), gastrointestinal disorders (11.5%), skin and subcutaneous tissue disorders (8.7%), infections and infestations (5.0%), musculoskeletal and connective tissue disorders (5.0%), respiratory, thoracic, and mediastinal disorders (4.0%), and injury, poisoning, and procedural complications (3.5%).

In our study, both Spikevax and Comirnaty demonstrated the same order of estimated reporting rates across ADR categories. The most often reported categories were neuropsychiatric disorders, cardiovascular disorders, gastrointestinal disorders, musculoskeletal and connective tissue disorders, respiratory, thoracic, and mediastinal disorders, and skin and subcutaneous tissue disorders. The least commonly reported categories were congenital, familial, and genetic disorders; renal and urinary disorders; and immune system disorders.

When comparing our findings to those of Alessia Zinzi et al., both studies identified neuropsychiatric (or nervous system) disorders and gastrointestinal disorders among the most often reported categories, along with skin and subcutaneous tissue disorders [15]. However, in our study, respiratory, thoracic, and mediastinal disorders, as well as musculoskeletal and connective tissue disorders, were more commonly reported than in the study by Alessia Zinzi et al [15]. A notable difference is that their study included mild and severe ADRs, whereas ours focused exclusively on serious ADRs only (Supplementary Material S2). This could explain why gastrointestinal and skin-related adverse reactions appeared less often in our data. Additionally, differences in the age ranges of participants could contribute to the observed discrepancies. Furthermore, the study by Alessia Zinzi et al [15]. did not report data for the six least common categories included in our analysis, thereby highlighting the novel evidence provided by our study on these underreported categories.

### 5.5. Limitations and Future Directions

The observational nature of cross-sectional studies limits their ability to establish causality. While useful for identifying potential correlations, such studies cannot determine whether these reflect direct causal links or associations influenced by unmeasured variables [16,53,54]. Additionally, the lack of randomization hinders control of confounding factors that may affect observed relationships [16,53,54]. Further well-designed, prospective studies are needed to confirm findings presented in this work and better define the safety profile of COVID-19 vaccines in children.

In the context of ADRs to COVID-19 vaccines, reliance on spontaneous reporting systems like EudraVigilance introduces additional challenges [23,38]. These systems are prone to underreporting, as they depend on healthcare professionals and the public to voluntarily submit data. Estimates suggest that EudraVigilance includes only about 6–10% of relevant adverse drug reactions [40]. Nevertheless, it remains the most comprehensive and systematically maintained pharmacovigilance database available at the European level [38]. While the data presented may underestimate the true burden, the relative patterns and associations observed seem to remain valid and informative.

Further on, data on pediatric populations remain substantially more limited than in adults, which may constrain the robustness and generalizability of conclusions regarding vaccine safety in children.

Moreover, the design of cross-sectional studies makes them particularly susceptible to recall bias; participants may struggle to accurately associate vaccination with specific adverse effects, potentially skewing the results. To mitigate these biases, we have decided to focus on serious ADRs that are less often underreported than mild and moderate ADRs [40]. SADRs hold greater clinical significance compared to mild ADRs due to their potential for substantial harm and their critical impact on patient safety [40].

By their very nature, SADRs are less likely to be overlooked. They often require urgent and intensive clinical management, including hospitalization or admission to intensive care units, contributing to significant healthcare system costs. Unlike mild ADRs, which are typically transient or manageable, SADRs may lead to long-term impairment, a substantial decline in the patient’s quality of life, or even mortality. Consequently, healthcare professionals and pharmaceutical companies are mandated to promptly report SADRs, underscoring their importance as critical concerns in healthcare and pharmacovigilance. This rigorous reporting is essential for assessing the risk-benefit profile of medications and ensuring the safety of therapeutic interventions.

In addition to that, there were disparities in vaccine distribution (Figure 1). For instance, 35,358,556 doses of Comirnaty were administered compared to 1,985,787 doses of Spikevax, thus the estimated reporting rates of ADRs reported for both vaccines may be skewed due to the differences in the number of administered vaccine doses. Moreover, for Novavax, the third vaccine authorized for use in children within the European Union, only 566 doses were administered according to data from the ECDC Vaccine Tracker database [55]. Furthermore, no serious adverse drug reactions (SADRs) meeting the eligibility criteria for our study were identified [23,38].

However, it is important to note that data for Novavax may be underreported, highlighting the need for more comprehensive and detailed monitoring. Enhanced surveillance would facilitate a robust comparison of Novavax with Spikevax and Comirnaty in terms of safety and adverse reaction profiles.

Despite these limitations, our study benefits from a substantial sample size, which enhances the robustness of the findings. Additionally, the results align closely with those of previously published research, reinforcing their validity. Nevertheless, longitudinal investigations with randomized designs are necessary to validate correlations found in this study and establish a clearer understanding of the causal relationships between anti-COVID-19 vaccination and adverse reactions. These future efforts will be critical to ensuring vaccine safety.

## 6. Conclusions

Several SADRs were reported in children following COVID-19 vaccination. The estimated real-world reporting rates of SADRs associated with authorized mRNA COVID-19 vaccines seem to be rare among children (0.57%), highlighting the potential safety of these vaccines in this population. Additionally, the data suggest that Comirnaty (Pfizer-BioNTech) has a very similar safety profile compared to Spikevax (Moderna). The most commonly reported clinical categories of suspected SADRs were neuropsychiatric, cardiovascular and gastroenterological disorders. The most often reported SADRs encompassed headaches, myocarditis, episodes of syncope, dizziness and dyspnea. Further research is needed to better characterize these events and to comprehensively assess the overall balance between the potential risks and benefits of vaccination in this population.

## Figures and Tables

**Table 1:** General demographic characteristics of the study cohort.

Demographic Characteristics	Comirnaty	Spikevax
Count	5018	404
Primary Source Qualification: Healthcare Professional, n (%)	2531.0 (50.4)	234.0 (57.9)
Primary Source Qualification: Non Healthcare Professional, n (%)	2487.0 (49.6)	170.0 (42.1)
Patient Age Group: 12–17 Years, n (%)	4360.0 (86.9)	380.0 (94.1)
Patient Age Group: 0–1 Month, n (%)	56.0 (1.1)	14.0 (3.5)
Patient Age Group: 2 Months–2 Years, n (%)	64.0 (1.3)	4.0 (1.0)
Patient Age Group: 3–11 Years, n (%)	538.0 (10.7)	6.0 (1.5)
Patient Sex: Male, n (%)	2483.0 (49.5)	204.0 (50.5)
Patient Sex: Female, n (%)	2435.0 (48.5)	197.0 (48.8)
Patient Sex: Not Specified, n (%)	100.0 (2.0)	3.0 (0.7)

**Table 2:** The estimated real-world reporting rates of the 25 most common SADRs in the pediatric population, expressed as normalized rates per million ADR reports and adjusted using real-world trial-based scaling. Information on all analyzed ADRs (n = 207) and their categories (n = 12) is available in Supplementary Material S2 or can be accessed through an online web application downloadable as Supplementary Material S3.

Variable	Total Reported ADRs (n)	Comirnaty—Trial-Adjusted Rate (Per Million ADR Reports, 95% CI)	Spikevax—Trial-Adjusted Rate (Per Million ADR Reports, 95% CI)
All adverse drug reactions	11,802	5792 (4634–6950)	5671 (4536–6805)
Headache	915	437 (331–550)	592 (483–707)
Myocarditis	672	322 (244–405)	419 (342–501)
Syncope	542	265 (201–333)	279 (228–334)
Dizziness	442	217 (165–274)	206 (168–246)
Dyspnoea	413	199 (151–251)	239 (195–286)
Nausea	377	179 (136–226)	253 (206–302)
Vomiting	367	178 (135–224)	206 (168–246)
Loss of consciousness	248	120 (91–151)	140 (114–167)
Abdominal pain	243	120 (91–151)	106 (87–127)
Myalgia	235	111 (84–140)	166 (136–199)
Rash	214	110 (83–138)	40 (33–48)
Seizure	213	110 (83–138)	40 (33–48)
Tachycardia	200	101 (77–127)	60 (49–72)
Pain in extremity	186	89 (68–112)	113 (92–135)
Myopericarditis	178	79 (60–99)	193 (157–231)
Pericarditis	178	87 (66–109)	93 (76–111)
Arthralgia	175	81 (61–102)	146 (119–175)
Urticaria	156	77 (58–97)	73 (60–87)
Paraesthesia	149	74 (56–93)	60 (49–72)
Diarrhea	140	72 (55–91)	27 (22–32)
Lymphadenopathy	137	67 (51–85)	66 (54–79)
Palpitations	131	63 (48–80)	73 (60–87)
Arrhythmia	109	57 (43–71)	13 (11–16)
Hypoaesthesia	107	53 (41–67)	40 (33–48)
Cough	99	48 (37–61)	53 (43–64)

**Table 3:** Categories of estimated real-world reporting rates of SADRs in the pediatric population, expressed as normalized rates per million ADR reports and adjusted using real-world trial-based scaling. Data for all analyzed ADR reports (n = 207) and their 12 categories can be found in Supplementary Material S2 or accessed through an online web application available for download as Supplementary Material S3.

Category of Adverse Drug Reactions	Total Reported ADRs (n)	Comirnaty—Trial-Adjusted Rate (Per Million ADR Reports, 95% CI)	Spikevax—Trial-Adjusted Rate (Per Million ADR Reports, 95% CI)
Neuropsychiatric disorders category	2532	1239 (939–1559)	1263 (1031–1510)
Cardiovascular disorders category	1753	853 (647–1073)	938 (765–1121)
Gastroenterological disorders category	1047	514 (389–646)	505 (413–604)
Musculoskeletal and connective tissue disorders category	716	345 (262–435)	419 (342–501)
Respiratory, thoracic and mediastinal disorders category	705	343 (260–431)	379 (309–453)
Skin and subcutaneous tissue disorders category	639	315 (239–397)	286 (233–342)
Ear and eye disorders category	358	176 (134–222)	166 (136–199)
Hematooncological disorders category	288	144 (109–181)	106 (87–127)
Obstetrical and gynecological disorders category	272	137 (104–172)	86 (71–103)
Immune system disorders category	211	105 (79–132)	86 (71–103)
Renal and urinary disorders category	70	32 (24–40)	66 (54–79)
Congenital, familial and genetic disorders category	13	5 (4–6)	27 (22–32)

## Data Availability

The data supporting this article can be found within the article itself, its online Supplementary Materials, and the EudraVigilance (1) and ECDC “Vaccine Tracker” (2) databases. https://www.adrreports.eu/en/search.html (9 February 2025). https://vaccinetracker.ecdc.europa.eu/public/extensions/COVID-19/vaccine-tracker.html (9 February 2025).

## Supplementary Materials

The following supporting information can be downloaded at https://www.mdpi.com/article/10.3390/jcm14186542/s1: S1: Grouping of the primary categories of adverse drug reactions into final category; S2: Estimated real-world reporting rates of the most common SADRs and their categories in the paediatric population; S3: Web application with interactive graph; S4: STROBE statement.
