# Supplementary material for: Serious Adverse Drug Reactions to COVID-19 Vaccines in the Pediatric Population: A Retrospective, Cross-Sectional Study Utilizing the Eudravigilance Database for the European Economic Area
Source: J Clin Med. 2025 Sep 17;14(18):6542. doi: 10.3390/jcm14186542 (PMC12471053; doi:10.3390/jcm14186542)
Supplement: Supplementary file 1 [file jcm-14-06542-s001.zip › Supplementary Material S3 R1.html]

Adverse drug reactions


### Choose data to display by selecting/unselecting a checkbox

##### Next to each checkbox a description of an adverse drug reaction and its total count across all studied vaccines is displayed. All frequencies of potential serious adverse drug reactions (ADRs) are reported as trial-adjusted normalized number of ADRs per million administered vaccine doses to children.
